# A large linked data platform to inform the COVID-19 response in British Columbia: The BC COVID-19 Cohort.

**DOI:** 10.23889/ijpds.v7i3.2095

**Published:** 2022-08-25

**Authors:** James Wilton, Jalud Abdulmenan, Mei Chong, Ana Becerra, Michael Coss, Marsha Taylor, Ognjenka Djurdjev, Drona Rasali, Hind Sbihi, Mel Krajden, Alexandra Flatt, Seyed Ali Mussavi Rizi, Naveed Janjua

**Affiliations:** 1 British Columbia Centre for Disease Control; 2 Provincial Health Services Authority

## Objectives

The COVID-19 pandemic has necessitated access to large health system datasets to inform the public health response. To meet this need, the Provincial Health Services Authority and the British Columbia (BC) Ministry of Health collaborated to create a population-based platform that integrates COVID-19 datasets with sociodemographic and administrative health data.

## Approach

A BC COVID Data Library proof-of-concept was created as a cloud-based, dynamic platform composed of de-identified datasets. The BC COVID-19 Cohort (BCC19C) represents a subset composed of people accessing COVID-19 health services (e.g., testing, vaccination) and linked health histories. Provincial COVID-19 datasets are updated daily and include COVID-19 lab tests, case surveillance, vaccinations and hospitalizations/deaths. These can be linked to administrative data holdings for the BC population, which are updated weekly/monthly and include vital statistics, medications, hospital admissions, medical visits, among others. A patient matching algorithm creates unique patient keys that allows the same individual to be linked across datasets.

## Results

The BCC19C has been used provincially to 1) support ongoing surveillance, reporting, and modelling of COVID-19; 2) describe and characterize the epidemiology of COVID-19; and 3) inform acute care planning, public health interventions and health care services in BC. Ongoing and completed BCC19C analyses include assessment of vaccine safety, vaccine effectiveness, and characteristics associated with infection and severe outcomes; use of medical visit data for syndromic surveillance and monitoring of unintended outcomes of the pandemic (e.g., mental health visits); and characterization of long-COVID. Availability of linked administrative data holdings has been crucial for identifying non-COVID control groups, measuring sociodemographics and co-morbidities, and complementing COVID-19 datasets for more complete capture of health outcomes (e.g., deaths, hospitalizations).

## Conclusion

The large scope/breadth and timeliness of the linkable datasets integrated within the COVID Data Library and the BCC19C has supported the public health response in BC. Additional linkage to other data sources will further strengthen this data platform.

